# Development and validation of a multivariable prediction model in open abdomen patients for entero‐atmospheric fistula

**DOI:** 10.1111/ans.17512

**Published:** 2022-02-04

**Authors:** Adam T. Cristaudo, Kerry Hitos, Ronny Gunnarsson, Alan Decosta

**Affiliations:** ^1^ Sydney Medical School The University of Sydney Sydney Australia; ^2^ Department of Surgery, Westmead Research Centre for Evaluation of Surgical Outcomes Westmead Hospital Westmead Australia; ^3^ General Practice / Family medicine, School of Public Health and Community Medicine, Institute of Medicine, Sahlgrenska Academy University of Gothenburg Gothenburg Sweden; ^4^ Research, Education, Development & Innovation Primary Health Care Region Västra Götaland Götaland Sweden; ^5^ Primary Health Care Clinic for Homeless People Gothenburg Sweden; ^6^ College of Medicine & Dentistry James Cook University Cairns Queensland Australia

**Keywords:** acute care surgery, general surgery, ICU, trauma, trauma acute care

## Abstract

**Background:**

Laparostomy or Open Abdomen (OA) has matured into an effective strategy in the management of abdominal catastrophe. Single prognostic factors have been identified in a previous systematic review regarding entero‐atmospheric fistula (EAF). Unfortunately, no prognostic multivariable model for EAF exist. The aim was to develop and validate a multivariable prediction model from a retrospective cohort study involving three hospital's databases.

**Methods:**

Fifty‐seven variables were evaluated to develop a multivariable model. Univariate and multivariable logistic regression analyses were performed for on a developmental data set from two hospitals. Receiver operator characteristics analysis with area under the curve (AUC) and 95% confidence intervals (CI) were performed on the developmental data set (internal validation) as well as on an additional validation data set from another hospital (external validation).

**Results:**

Five‐hundred and forty‐eight patients managed with an OA. Two variables remained in the multivariable prediction model for EAF. The AUC for EAF on internal validation were 0.74 (95% CI: 0.58–0.86) and 0.79 (95% CI: 0.67–0.92) on external validation.

**Conclusions:**

A multivariable prediction model for EAF was externally validated and an easy‐to‐use probability nomogram was constructed using the two predictor variables.

Level of evidence: III; prognostic.

## Graphical abstract

The list of complications associated with open abdomen management is formidable. Of these, Entero‐Atmospheric Fistula (EAF) is the most feared, associated with mortality, cost and disability. This study produced a simple, yet usable, prediction model for EAF that is internally and externally validated and identifies Aboriginal and/or Torres Strait Islander status and the number of laparotomies as predictive of EAF.

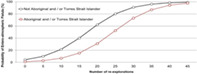

## Introduction

### Background and objectives

Laparostomy or Open Abdomen (OA) has matured into an effective strategy in the management of abdominal catastrophe.^1^

The list of complications associated with OA management is formidable. Of these, Entero‐Atmospheric Fistula (EAF) is the most feared, associated with mortality, cost and disability. While the notion of OA suggests an accessible abdomen, repeated visceral manipulation remains a considerable hazard best avoided.^2^

The management of EAF is well described in the current literature; however preventative methods are less described.^3^ Failed definitive fascial closure (DFC), large bowel resection and total fluid intake at 48 h were identified as single prognostic factors regarding the development of an EAF.^4^, ^5^ Currently there are no prognostic multivariable models regarding EAF. The objective of this study was to develop and validate a multivariable prediction model for EAF from a retrospective cohort involving three hospital's databases.

## Methods

This study first used a development data set for constructing a multivariable prediction model and internally validate it. A second validation data set was then used to externally validate the multivariable prediction model. Ethics approval was provided by the Townsville Hospital and Health Services Human Research Ethics Committee (Reference: HREC/15/QTHS/1).

### Source of data

Data from Cairns and Townville Hospitals in Australia were used to form the developmental data set, whilst data from the Royal Brisbane and Women's Hospital in Australia were used for the validation data set. Patients being admitted between 1st of January 2000 to 19th December 2016 were included.

Observations were sourced from Operating Room Management Information System (ORMIS; January 2000 to November 2012 – ORMIS version 5; November 2012 to March 2016 – ORMIS version 7) case validation reports and Surgi‐Net (for data from March 2016 to December 2016). Cross‐checking was performed using medical records ‐ both electronic (i.e., The Viewer) and paper‐based – with ORMIS and Surgi‐Net data. Laboratory results were obtained from AUSLAB.

### Participants

Patients aged 18 years and above, who underwent a midline laparotomy, regardless of indication or sex, and were unable to have primary definitive fascial closure completed at the end of the case which necessitated temporary abdominal closure were eligible for inclusion. Patients who had their ‘index operation’ prior to presenting to either of the three sites were excluded. All OA patients were managed in line with current guidelines and according to the treating consultant's preference.^6^

### Outcomes

The outcome EAF was confirmed from progress notes from their index admission from either paper‐based or electronic medical records. EAF was defined as the development of an abnormal communication between the gastrointestinal tract and the ‘atmosphere’ without overlying soft tissue of a patient being managed with an OA at any time after initial laparostomy. No actions were taken to blind assessment of the outcomes to be predicted.

### Predictors

Potential predictors evaluated in the development of the multivariable prediction model stemmed from a recently published systematic review.^3^ These predictors were defined in detail before commencing data collection (Table S1). No actions were taken to blind assessment of potential predictors for relevant outcomes.

### Missing data

Prognostic factors with more than 20% of patient data missing (Anaesthesiologist's Society of America (ASA) score; physiology and operative severity score, morbidity and mortality (%) (from P‐POSSUM score); body mass index (BMI); systolic blood pressure; pulse rate; Glasgow coma scale (GCS); weight; days until enterally fed; use of total parenteral nutrition and enteral nutrition) were excluded from subsequent logistic regression analyses and modelling. These prognostic factors had missing data due to apparent lack of documentation (i.e., ASA score), due to them not being measured (i.e., weight), or due to their inability of being calculated due to incomplete data (i.e., BMI, P‐POSSUM scoring values). The outcome EAF was clearly defined and hence no data were missing. No imputation methods were required or used.

### Sample size

Sample size calculations were based on significant prognostic factors from the recently published systematic review regarding each of the outcomes.^3^ All sample size calculations were performed using the software G*Power version 3.1.9.2 with the level of significance set to 0.05, the power to 95% and using a two‐tailed test.^7^

The sample sizes required for analysing the different independent prognostic factors were for (a) large bowel resection: 287 patients; for (b) failed delayed fascial closure: 99 patients.^4^, ^5^ Therefore, for the expected number of significant variables considered within our study, the aim is to include a total of at least 287 patients.

### Statistical analysis methods

Predictors were handled as either continuous or dichotomous (categorical) variables in the analysis. The outcome EAF was handled as a binary variable. Normality was tested using Kolmogorov–Smirnov and Shapiro–Wilk tests to evaluate all distributions.

The development and validation data set were compared using chi‐square test for categorical variables and the student's *t*‐test for independent continuous variables with normal distribution. For non‐parametric data, the Mann Whitney test was used. Non‐parametric continuous variables were presented as median values and interquartile range (IQR: 25th and 75th percentile). All tests were two tailed and the level of significance was set to <0.05.

Univariate logistic regression analyses using the developmental data set were performed for all prognostic factors to identify statistically significant (*p* < 0.05) and other potentially relevant (*p* < 0.2) factors. These (both statistically significant and other potentially relevant) prognostic factors were included in a subsequent multivariable backwards stepwise logistic regression analysis. The final model was built based on the prognostic factors that remained statistically significant in the multivariable model. The results were presented as odds ratio (OR) with 95% confidence intervals (CI).

The developmental data set from Cairns and Townsville Hospitals were also used for internal validation of the new multivariable prediction model. External validation was then performed using the validation data set from Royal Brisbane and Women's Hospital. Only the independent predictor variables identified from the development data set were used in the external validation. Models were assessed for performance according to receiver operator characteristics (ROC) analysis with area under curve (AUC) with their relevant 95% CI and p‐value.

Statistical analyses were performed using International Business Machines (IBM) Corporation Statistical Packages for Social Sciences (SPSS) version 24.0 and version 26.0.^8^, ^9^

### Results

The development data set had 312 patients where 23 (7.4%) developed an EAF while the validation data set had 236 patients where 23 (9.7%) developed EAF (*p* = 0.89). Apart from hospital location and catchment area there were no differences between the development data set and the validation data set in terms of eligibility criteria, outcomes and predictors (refer to Tables S2–S6).

### Model development

In developing the model for EAF (using the developmental data set), six predictive variables were statistically significant (*p* < 0.05), and six others were potentially interesting (*p* < 0.20) of the 57 included in the univariate logistic regression analysis (Table 1). Of these, nine independent predictive variables were included in the stepwise multivariable logistic regression. Three predictive variables were excluded due to less than 80% of data being available. Two (Aboriginal and/or Torres Strait Islander status and number of re‐explorations) remained in the internal validation model (Table 1).

**Table 1:** Associations between each candidate predictor and entero‐atmospheric fistula for internal validation model

	Univariate analysis (*n* = 312)					Multivariable analysis (*n* = 232)				
Prognostic factors	*n*	*B*	*P*	OR	95% CI	*n*	*B*	*P*	OR	95% CI
Diagnosis										
Intra‐abdominal sepsis	312	−0.26	0.56	0.77	0.32–1.9					
Perforation	312	−0.38	0.55	0.68	0.20–2.4					
Ischaemic bowel	312	0.19	0.75	1.2	0.39–3.7					
Post‐operative haemorrhage	312	−19	1.0	7.5 × 10^−9^	0‐∞					
Peritonitis	312	−0.34	0.66	0.71	0.16–3.2					
Intestinal obstruction	312	0.55	0.48	1.7	0.37–8.1					
Anastomotic leak	312	4.8 ×10^−2^	0.96	1.0	0.13–8.4					
Malignancy	312	0.14	0.90	1.1	0.14–9.3					
Sepsis (other)	312	−19	1.0	7.5 × 10^−9^	0‐∞					
Necrotising fasciitis	312	1.2	0.30	3.2	0.35–30					
Trauma	312	−1.2	0.26	0.26	4.1 × 10^−2^‐2.4					
**Severe acute pancreatitis**	**312**	**1.4**	**0.006**	**3.9**	**1.5–10**	–	–	–	–	–
Vascular surgery	312	−0.38	0.72	0.68	8.7 × 10^−2^‐5.4					
Intra‐abdominal hypertension/abdominal compartment syndrome	312	−19	1.0	7.5 × 10^−9^	0‐∞					
Abdominal compartment syndrome	312	−19	1.0	7.5 × 10^−9^	0‐∞					
Wound dehiscence	312	−19	1.0	7.7 × 10^−9^	0‐∞					
Age	312	2.8 × 10^−3^	0.83	1.00	0.977–1.03					
Age (>61years)	312	0.26	0.55	1.3	0.55–3.0					
Sex (Male)	312	0.11	0.81	1.1	0.46–2.7					
**B**										
Bowel resection										
Before index operation	312	−0.79	0.30	0.46	0.10–2.0					
At index operation	312	−0.13	0.78	0.88	0.36–2.1					
**After index operation**	**312**	**0.73**	**0.10**	**2.1**	**0.88–4.9**	–	–	–	–	–
APACHE III Score	312	−3.5 × 10^−3^	0.62	1.00	0.983–1.01					
Evidence of cardiac disease	268	−0.25	0.59	0.78	0.31–1.9					
Evidence of respiratory disease	268	0.34	0.46	1.4	0.56–3.5					
**Electrocardiogram**	**269**	**−1.4**	**0.17**	**0.24**	**3.1** × **10** ^**−2**^ **‐1.8**	–	–	–	–	–
Haemoglobin	312	−5.6 × 10^−2^	0.46	1.0	0.81–1.1					
White blood cell count	312	−1.0 × 10^−2^	0.68	0.99	0.94–1.0					
**Urea**	**312**	**−9.4** × **10** ^**−2**^	**0.073**	**0.91**	**0.82–1.0**	–	–	–	–	–
Sodium	312	−1.2 × 10^−2^	0.76	1.0	0.92–1.1					
Potassium	312	−0.30	0.33	0.74	0.41–1.3					
Operation type	307	‐	‐	‐	‐					
Operative blood loss	304	0.31	0.49	1.4	0.56–3.3					
Peritoneal contamination	303	0.25	0.74	1.3	0.29–5.8					
Malignancy status	305	−19	1.0	7.2 × 10^−9^	0‐∞					
CEPOD classification of intervention	312	0.23	0.76	1.3	0.28–5.6					
**Serum albumin**	**312**	**−7.4** × **10** ^**−2**^	**0.04**	**0.93**	**0.87–1.0**	–	–	–	–	–
Topical negative pressure	312	−0.42	0.33	0.66	0.28–1.5					
**Aboriginal and/or Torres Strait islander status**	**312**	**−1.1**	**0.16**	**0.35**	**8.0** × **10** ^**−2**^ **‐1.5**	**232**	**−1.3**	**0.12**	**0.27**	**5.1** × **10** ^**−2**^ **‐1.4**
**C**										
Days abdomen open**	**219**	**0.15**	**0.0002**	**1.2**	**1.1–1.3**					
**Number of procedures**	**307**	**0.16**	**<0.0001**	**1.2**	**1.1–1.3**	–	–	–	–	–
**Study year**	**312**	**−5.8** × **10** ^**−2**^	**0.20**	**0.94**	**0.86–1.0**	–	–	–	–	–
**Number of re‐explorations**	**307**	**0.16**	**<0.0001**	**1.2**	**1.1–1.3**	**232**	**0.18**	**<0.0001**	**1.2**	**1.1–1.3**
Not closed (failed definitive fascial closure)	312	3.2 × 10^−2^	0.95	1.0	0.41–2.6					
ASA Score**	**160**	**0.73**	**0.069**	**2.1**	**0.95–4.5**					
Physiology Score**	152	−5.8 × 10^−2^	0.22	0.94	0.86–1.0					
Operative Severity Score**	152	2.2 × 10^−2^	0.68	1.0	0.92–1.1					
Morbidity (%)**	152	−1.5 × 10^−3^	0.96	1.0	0.94–1.1					
Mortality (%)**	152	−9.3 × 10^−3^	0.44	0.99	0.97–1.0					
Body Mass Index**	82	1.7 × 10^−2^	0.83	1.0	0.87–1.2					
Systolic blood pressure**	155	6.0 × 10^−3^	0.57	1.01	0.985–1.03					
Pulse rate**	154	1.0 × 10^−3^	0.96	1.00	0.975–1.03					
Glasgow coma scale**	162	−7.5 × 10^−2^	0.40	0.93	0.78–1.1					
Weight**	151	−1.8 × 10^−2^	0.30	0.98	0.95–1.0					
Days until enterally fed**	91	−0.43	0.28	0.65	0.30–1.4					
Total parenteral nutrition**	**206**	**1.4**	**0.007**	**4.0**	**1.5–11**					
Enteral feeding**	207	−0.23	0.66	0.80	0.29–2.2					

*Note*: NB: Constant (B): −3.1 (from stepwise multivariable logistic regression); **, incomplete variable data set (<80% of data available; NOT included in multivariable analysis. Bold values are significant findings, p < 0.05.

**Fig. 1:**
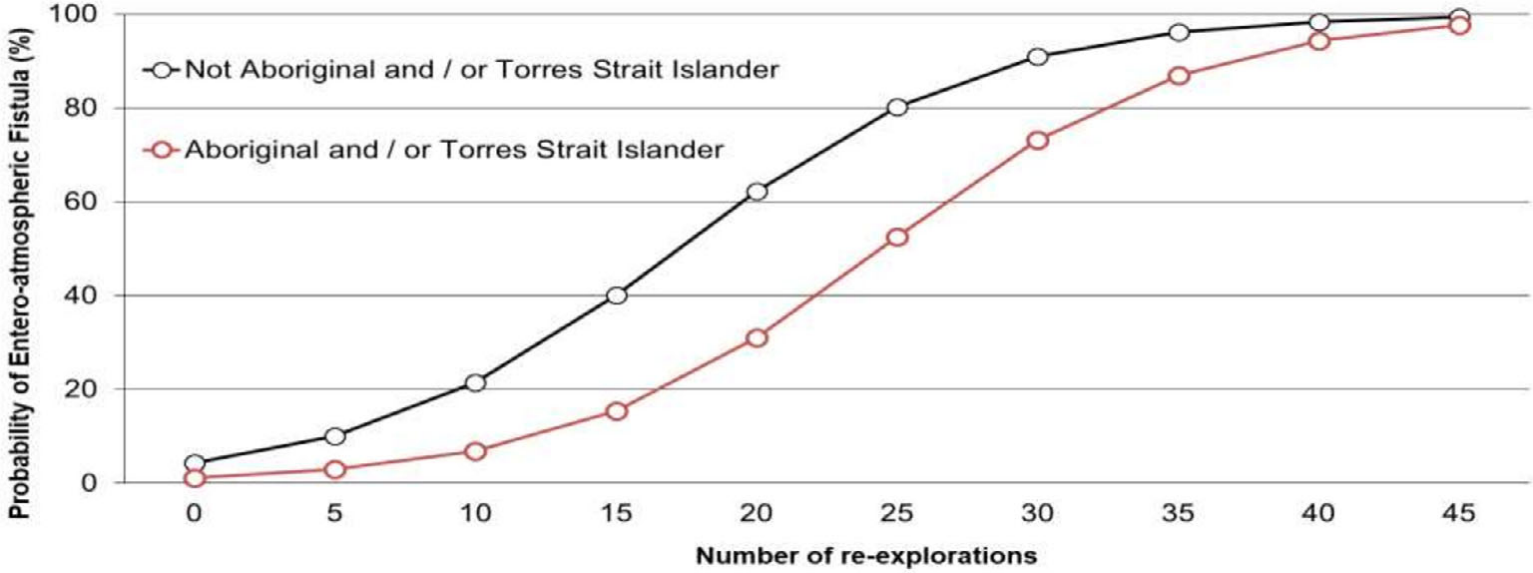
Probability nomogram for the multivariable prediction model for entero‐atmospheric fistula.

The final prediction model for EAF included one independent continuous predictor (number of procedures) and one binary (Aboriginal and/or Torres Strait Islander status). This allowed the model to be visualized in the form of a probability nomogram (Fig. 1).

### Internal and external validation

Due to some missing data the internal validation included 269 patients and the external validation 232 patients. AUC at the internal validation was 0.74 (95% CI: 0.58–0.86, *p* = 0.0001) and at the external validation 0.74 (95% CI 0.67–0.92, *p* < 0.0001).

## Discussion

This study produced a simple, yet usable, multivariable prediction model for EAF.

### Methodological aspects

In terms of strengths, more potential prognostic factors have been evaluated in this retrospective cohort study than any previous studies.^3^ This and the total number of patients included (312 + 236 = 548) is also a strong point in this study with the data set extending over 17 years of OA management and three tertiary hospitals. Another strength of this study is that both internal as well as external validation was done in 501 (269 + 232) patients with very similar results.

Missing data from medical records and operative notes were minimalized by meticulous cross‐checking where available. Despite this, a few potential predictor variables had to be excluded from the multivariable logistic regression due to >20% missing data. This incurs a small risk of confounding the results.

The management of the OA has changed over the years. Initial approaches to managing patients with an open abdomen included a planned ventral hernia approach, with little or no temporary abdominal closure techniques in place. The present standard of care mandates forms of negative pressure therapy aimed at keeping the abdomen open for the shortest time possible.^10^ This aids in improving chances of definitive fascial closure whilst decreasing incidence of complications. These differences in management approach allow for varying standards of care amongst this cohort of patients; the potential effect this may have had on the outcomes in this study is immeasurable, but worth mentioning.

### Interpretation

These findings highlight the importance of such a study, 8.4% of patients developed an EAF. This shows the inherent need to reduce complications for patients being managed with an OA in terms of EAF alone.

Although no previous multivariable prediction models for EAF exists, previous studies have identified single factors being associated with increased risk for developing EAF. These previously identified factors; failed DFC, large bowel resection and total fluid intake at 48 h of 5–10 L and >10 L, differed from the factors identified in our multivariable model. Our study evaluated more potential prognostic factors and included more patients than previous studies. This multivariable prediction model is internally and externally validated and identifies Aboriginal and/or Torres Strait Islander status and the number of laparotomies as predictive of EAF.

### Authorship statement

The first author (Dr Adam T Cristaudo) was responsible for all aspects of this submission, including the research questions, hypothesis and aims, the data collection process, database design, data entry, retrievals and/or extraction, statistical analysis and write up. Study concept, design, statistical analysis and writing of manuscript was carried out in collaboration with the other listed authors A/Prof. Kerry Hitos, Prof. Ronny Gunnarsson and A/Prof. Alan de Costa. All authors approved the final version of the manuscript.

## Author contributions

**Adam Thomas Cristaudo:** Conceptualization; data curation; formal analysis; investigation; methodology; project administration; resources; software; validation; visualization; writing – original draft; writing – review and editing. **Kerry Hitos:** Conceptualization; formal analysis; investigation; methodology; project administration; resources; software; supervision; validation; visualization; writing – review and editing. **Ronny Gunnarsson:** Conceptualization; formal analysis; investigation; methodology; project administration; resources; software; supervision; validation; visualization; writing – review and editing. **Alan de Costa:** Conceptualization; formal analysis; investigation; methodology; project administration; resources; software; supervision; validation; visualization; writing – review and editing.

## Conflicts of interest

None declared.

## Supporting information


**Table S1.A** Predictors: Definitions, includes how and when measured
**Table S1.B**: Predictors: Definitions, includes how and when measured
**Table S1.C**: Predictors: Definitions, includes how and when measured
Table S1.D: Predictors: Definitions, includes how and when measured
Table S1.E: Predictors: Definitions, includes how and when measured
**Table S1.F**: Predictors: Definitions, includes how and when measured
Table S1.G: Predictors: Definitions, includes how and when measured
**Table S1.H**: Predictors: Definitions, includes how and when measured
**Table S2**: Comparison of prognostic factors between developmental and validation data sets: Demographics
**Table S3**: Comparison of prognostic factors between developmental and validation data sets: Nutrition
**Table S4**: Comparison of prognostic factors between developmental and validation data sets: Diagnosis
**Table S5.A**: Comparison of prognostic factors between developmental and validation data sets: Clinical scoring systems
**Table S5.B**: Comparison of prognostic factors between developmental and validation data sets: Clinical scoring systems
**Table S6**: Comparison of prognostic factors between developmental and validation data sets: Interventions
