# Supplementary material for: Development and validation of a multivariable prediction model in open abdomen patients for entero‐atmospheric fistula
Source: ANZ J Surg. 2022 Feb 4;92(5):1079–84. doi: 10.1111/ans.17512 (PMC9303347; doi:10.1111/ans.17512)
Supplement: Supplementary file 1 — Table S1.A Predictors: Definitions, includes how and when measured Table S1.B: Predictors: Definitions, includes how and when measured Table S1.C: Predictors: Definitions, includes how and when measured Table S1.D: Predictors: Definitions, includes how and when measured Table S1.E: Predictors: Definitions, includes how and when measured Table S1.F: Predictors: Definitions, includes how and when measured Table S1.G: Predictors: Definitions, includes how and when measured Table S1.H: Predictors: Definitions, includes how and when measured Table S2: Comparison of prognostic factors between developmental and validation data sets: Demographics Table S3: Comparison of prognostic factors between developmental and validation data sets: Nutrition Table S4: Comparison of prognostic factors between developmental and validation data sets: Diagnosis Table S5.A: Comparison of prognostic factors between developmental and validation data sets: Clinical scoring systems Table S5.B: Comparison of prognostic factors between developmental and validation data sets: Clinical scoring systems Table S6: Comparison of prognostic factors between developmental and validation data sets: Interventions [file ANS-92-1079-s001.docx]

#### Table S1.A: Predictors: Definitions, includes how and when measured

| **Predictor** | **Definition** | **How measured** | **When measured** |
| --- | --- | --- | --- |
| Nutrition |  | | |
| Serum albumin | Patient’s albumin level (normal range: 35-55 g/L) | From pathology (AUSLAB) | Immediately prior to initial laparostomy |
| Days until enterally fed | Days until patient was enterally fed (either orally or via tube) | From medical records – Dietician assessment | During index admission, after initial laparostomy |
| Total Parenteral Nutrition | Parenteral based nutrition given to the patient at any stage after initial laparostomy | From medical records – Dietician assessment | During index admission, after initial laparostomy |
| Enteral feeding | Enteral based nutrition initiated at any stage following patient’s initial laparostomy | From medical records – Dietician assessment | During index admission, after initial laparostomy |
| Diagnosis |  | | |
| Intra-Abdominal Sepsis | As underlying diagnosis leading to an open abdomen | Based on admission notes | At time of index admission |
| - Perforation (of viscous) | As underlying diagnosis leading to an open abdomen | Based on admission notes | At time of index admission |
| - Ischaemic bowel | As underlying diagnosis leading to an open abdomen | Based on admission notes | At time of index admission |
| - Post-operative haemorrhage | As underlying diagnosis leading to an open abdomen | Based on admission notes | At time of index admission |

#### Table S1.B: Predictors: Definitions, includes how and when measured

| **Predictor** | **Definition** | **How measured** | **When measured** |
| --- | --- | --- | --- |
| Diagnosis (continued…) |  | | |
| - Peritonitis | As underlying diagnosis leading to an open abdomen | Based on admission notes | At time of index admission |
| - Intestinal obstruction | As underlying diagnosis leading to an open abdomen | Based on admission notes | At time of index admission |
| - Anastomotic leak | As underlying diagnosis leading to an open abdomen | Based on admission notes | At time of index admission |
| - Malignancy | As underlying diagnosis leading to an open abdomen | Based on admission notes | At time of index admission |
| - Sepsis | As underlying diagnosis leading to an open abdomen | Based on admission notes | At time of index admission |
| - Necrotising fasciitis | As underlying diagnosis leading to an open abdomen | Based on admission notes | At time of index admission |
| Trauma | As underlying diagnosis leading to an open abdomen | Based on admission notes | At time of index admission |
| Severe Acute Pancreatitis | As underlying diagnosis leading to an open abdomen | Based on admission notes | At time of index admission |
| Vascular surgery | As underlying diagnosis leading to an open abdomen | Based on admission notes | At time of index admission |

#### Table S1.C: Predictors: Definitions, includes how and when measured

| **Predictor** | **Definition** | **How measured** | **When measured** |
| --- | --- | --- | --- |
| Diagnosis (continued…) |  |  |  |
| Intra-abdominal hypertension | Intra-abdominal pressure ≥12mmHg leading to open abdomen | Based on ICU notes | At time of index admission |
| - Abdominal compartment syndrome | Intra-abdominal pressure >20mmHg leading to open abdomen | Based on ICU notes | At time of index admission |
| - Wound dehiscence | As underlying diagnosis leading to an open abdomen | Based on admission notes | At time of index admission |
| Demographics  (pre-operative) |  | | |
| Age | Patients age in years | Based on date of birth | At initial laparostomy |
| Age > 61 years (from P-POSSUM score) | Patients aged > 61 years | Based on date of birth | At initial laparostomy |
| Sex | Male or female | Medical record details | At time of index admission |
| Aboriginal and / or Torres Strait Islander | Patient identifies as Aboriginal and / or Torres Strait Islander | From medical records | At index admission |
| Study year | Calendar year initial laparostomy performed | From medical records | At initial laparostomy |

#### Table S1.D: Predictors: Definitions, includes how and when measured

| **Predictor** | **Definition** | **How measured** | **When measured** |
| --- | --- | --- | --- |
| Anthropometry |  | | |
| Body Mass Index | Dietician assessment of patient’s height and weight to form their body mass index (weight (in kilograms)/height^2^(in metres) | From medical records | During index admission, prior to initial laparostomy |
| Weight | Dietician assessment of patient’s weight (in kilograms) | From medical records | During index admission, prior to initial laparostomy |
| Clinical Scoring Systems |  | | |
| Acute Physiology & Chronic Health Evaluation (APACHE) III score | Mortality risk stratification tool (range 0-299) | From ICU data manager | On admission to the ICU |
| American Society of Anaesthesiologist (ASA) Score | Classification system of physical fitness status of patients | From operative notes | Immediately prior to initial laparostomy |
| Physiology Score (from P-POSSUM) | Physiological risk score predicting morbidity and mortality | From P-POSSUM scores | Immediately prior to initial laparostomy |
| Operative Severity Score (from P-POSSUM) | Operative risk score predicting morbidity and mortality | From P-POSSUM scores | Immediately prior to initial laparostomy |
| Morbidity (%) (from P-POSSUM) | Percentage risk of patient morbidity | From P-POSSUM scores | Immediately prior to initial laparostomy |

#### Table S1.E: Predictors: Definitions, includes how and when measured

| **Predictor** | **Definition** | **How measured** | **When measured** |
| --- | --- | --- | --- |
| Clinical Scoring Systems (continued…) |  | | |
| Mortality (%) (from P-POSSUM) | Percentage risk of patient mortality | From P-POSSUM scores | Immediately prior to initial laparostomy |
| Interventions |  | | |
| Bowel Resection |  |  |  |
| - Before Index Operation | Bowel resected at any time before initial laparostomy | From operative notes | Before initial laparostomy |
| - At Index Operation | Bowel resected at initial laparostomy | From operative notes | At initial laparostomy |
| - After Index Operation | Bowel resected at any time after initial laparostomy | From operative notes | After initial laparostomy |
| Operation Type | Complexity of operation (minor, major, complex major) | From operative notes | At initial laparostomy |
| Operative Blood Loss | Estimated blood loss in mL (significant: any value > 100mL) | From operative notes | At initial laparostomy |
| Topical Negative Pressure | Type of temporary abdominal closure technique involving negative pressure wound therapy | From operative notes | At initial laparostomy |
| Days abdomen open | Days until definitive fascial closure was achieved | From operative notes | During index admission |
| Number of procedures | Number of laparotomies after and including initial laparostomy | From operative notes | During index admission |

#### Table S1.F: Predictors: Definitions, includes how and when measured

| **Predictor** | **Definition** | **How measured** | **When measured** |
| --- | --- | --- | --- |
| Pre-operative Parameters |  | | |
| Electrocardiogram (ECG) | Presence of any ECG abnormalities (i.e., AF, rate 60-90; >4/min ectopics; Q waves; ST/T changes; any other abnormal rhythm) | From medical records | Immediately prior to initial laparostomy |
| Systolic Blood Pressure | Patient’s systolic blood pressure (normal range 110-130 mmHg) | From admission notes | Immediately prior to initial laparostomy |
| Pulse Rate | Patient’s pulse rate as assessed by staff (normal range: 50-80 beats per minute) | From admission notes | Immediately prior to initial laparostomy |
| Haemoglobin | Patient’s red blood cell count (normal range: 13-16 g/dl) | From AUSLAB | Immediately prior to initial laparostomy |
| White blood cell count | Patient’s white blood cell count (normal range: 4-10 mmol/L) | From AUSLAB | Immediately prior to initial laparostomy |
| Urea | Patient’s urea level (normal range: < 7.6 mmol/L) | From AUSLAB | Immediately prior to initial laparostomy |
| Sodium | Patient’s sodium level (normal range: >135 mmol/L) | From AUSLAB | Immediately prior to initial laparostomy |
| Potassium | Patient’s potassium level (normal range: 3.5-5.0 mmol/L) | From AUSLAB | Immediately prior to initial laparostomy |

#### Table S1.G: Predictors: Definitions, includes how and when measured

| **Predictor** | **Definition** | **How measured** | **When measured** |
| --- | --- | --- | --- |
| Pre-operative Parameters (continued…) |  | | |
| Glasgow Coma Scale | Method of assessment of a patient’s impairment of conscious level in response to defined stimuli (normal: 15) | From admission notes | Immediately prior to initial laparostomy |
| CEPOD Classification of Intervention | Elective (planned) vs. emergency theatre case | From operative notes | At initial laparostomy |
| Co-morbidities (pre-morbid) |  | | |
| Evidence of cardiac disease | Presence of any cardiac disease (i.e., diuretic, digoxin, treatment for angina or hypertension; peripheral oedema, warfarin, cardiomyopathy; raised jugular venous pressure, cardiomegaly) | From medical records | Immediately prior to initial laparostomy |
| Evidence of respiratory disease | Presence of any symptoms of respiratory disease (i.e., dyspnoea (on exertion, limiting or at rest); chronic obstructive airways disease (mild or moderate); pulmonary fibrosis/consolidation on x-ray) | From medical records | Immediately prior to initial laparostomy |
| Complications |  | | |
| Peritoneal Contamination | Soiling of the peritoneal cavity (minor, local pus, free pus / blood / bowel content) | From operative notes | At initial laparostomy |

#### Table S1.H: Predictors: Definitions, includes how and when measured

| **Predictor** | **Definition** | **How measured** | **When measured** |
| --- | --- | --- | --- |
| Complications (continued…) |  | | |
| Malignancy Status | Any evidence of malignancy noted | From operative notes | At initial laparostomy |

#### Table S2: Comparison of prognostic factors between developmental and validation data sets: Demographics

| **Prognostic factor** | **Developmental data set**  **(n = 312)** | | **Validation data set**  **(n = 236)** | | **Statistical result** |
| --- | --- | --- | --- | --- | --- |
|  | n | % | n | % | p-value |
| Male sex | 196 | 63 | 147 | 62 | 0.55 |
| Aboriginal and / or Torres Strait Islander | 64 | 20 | 10 | 4.3 | 0.72 |
|  | median value and interquartile range (IQR) | | median value and IQR | | p-value |
| Body mass index (BMI; kg/m^2^) | 28 (IQR: 18 to 38) | | N/A | | N/A |
| Weight (kg) | 79 (IQR: 55 to 103) | | N/A | | N/A |
| Age (years) | 59 (IQR: 34 to 84) | | 56 (IQR: 29 to 83) | | 0.05 |
| Study year | 2010 (IQR: 2003 to 2017) | | 2010 (IQR: 2002 to 2018) | | <0.0001^ |

Chi-square test used for categorical variables; Mann Whitney test was used for non-parametric continuous dependent variables; ^Statistically significant (p <0.05)

#### Table S3: Comparison of prognostic factors between developmental and validation data sets: Nutrition

| **Prognostic factor** | **Developmental data set**  **(n = 312)** | | **Validation data set**  **(n = 236)** | | **Statistical result** |
| --- | --- | --- | --- | --- | --- |
|  | n | % | n | % | p-value |
| Total parenteral nutrition (used during index admission) | 64/206 | 31 | N/A | N/A | N/A |
| Enteral nutrition (via feeding tube; used during index admission) | 132/207 | 64 | N/A | N/A | N/A |
|  | median value and interquartile range (IQR) | | median value and IQR | | p-value |
| Serum albumin (normal range: 35-55 g/L) | 24 (IQR: 13 to 35) | | N/A | | N/A |
| Days until enterally fed (either orally or via feeding tube) | 2 (IQR: 0 to 4) | | N/A | | N/A |

Chi-square test used for categorical variables; Mann Whitney test was used for non-parametric continuous dependent variables.

#### Table S4: Comparison of prognostic factors between developmental and validation data sets: Diagnosis

| **Prognostic factor** | **Developmental data set**  **(n = 312)** | | **Validation data set**  **(n = 236)** | | **Statistical result** |
| --- | --- | --- | --- | --- | --- |
|  | n | % | n | % | p-value |
| Intra-abdominal sepsis | 207 | 66 | 146 | 62 | 0.45 |
| Trauma | 38 | 12 | 64 | 27 | 0.80 |
| Severe acute pancreatitis | 36 | 12 | 14 | 6.0 | 0.23 |
| Vascular surgery | 19 | 6.2 | 2 | 0.8 | 1.00 |
| Intra-abdominal hypertension / Abdominal compartment syndrome | 12 | 3.8 | 10 | 4.2 | 1.00 |

Chi-square test used for categorical variables

#### Table S5.A: Comparison of prognostic factors between developmental and validation data sets: Clinical scoring systems

| **Prognostic factors** | **Developmental data set**  **(n = 312)** | | **Validation data set**  **(n = 236)** | | **Statistical result** |
| --- | --- | --- | --- | --- | --- |
|  | n | % | n | % | p-value |
| Physiological parameters  (from P-POSSUM) |  | | | | |
| Evidence of cardiac disease | 117/268 | 44 | 72/181 | 40 | 1.0 |
| Evidence of respiratory disease | 83/268 | 31 | 66/181 | 36 | 0.42 |
| Abnormal electrocardiogram | 44/269 | 16 | 17/181 | 9.4 | 1.0 |
| Operative parameters  (from P-POSSUM) |  | | | | |
| Operation type  (major / complex major) | 307/307 | 100 | N/A | N/A | N/A |
| Operative blood loss (> 100 ml) | 104/304 | 34 | N/A | N/A | N/A |
| Peritoneal contamination  (at initial laparostomy) | 269/303 | 89 | 199/234 | 85 | 1.0 |
| Malignancy status  (at initial laparostomy) | 25/305 | 8.2 | 20/233 | 8.6 | 0.26 |
| CEPOD classification of intervention (emergency cases) | 279 | 89 | 208 | 88 | 1.0 |
|  | median value and interquartile range (IQR) | | median value and IQR | | p-value |
| Clinical scoring systems |  | | | | |
| APACHE III score (range: 0-299) | 70 (IQR: 32 to 108) | | 66 (IQR: 27 to 105) | | 0.06 |
| ASA score (range: 1-5) | 4 (IQR: 3 to 5) | | N/A | | N/A |
| Morbidity (%; from P-POSSUM) | 95 (IQR: 81 to 100) | | N/A | | N/A |
| Mortality (%; from P-POSSUM) | 29 (IQR: 0 to 73) | | N/A | | N/A |
| Physiology score  (range:12-88; from P-POSSUM) | 24 (IQR: 12 to 35) | | N/A | | N/A |

#### Table S5.B: Comparison of prognostic factors between developmental and validation data sets: Clinical scoring systems

| **Prognostic factors** | **Developmental data set**  **(n = 312)** | **Validation data set**  **(n = 236)** | | **Statistical result** | |
| --- | --- | --- | --- | --- | --- |
|  | n | % | | n | |
| Operative severity score  (range: 6-48; from P-POSSUM) | 26 (IQR: 18 to 34) | N/A | | N/A | |
| Physiological parameters  (from P-POSSUM) |  | | | | |
| Systolic blood pressure (mmHg) | 125 (IQR: 87 to 163) | | N/A | | N/A |
| Pulse rate (beats per minute) | 100 (IQR: 67 to 133) | | N/A | | N/A |
| White blood cell count (mmol/L) | 13.1 (IQR: 2.9 to 23) | | 12.5 (IQR: 2.4 to 23) | | 0.45 |
| Haemoglobin (g/dl) | 12.0 (IQR: 7.7 to 16) | | 10.2 (IQR: 5.7 to 15) | | 0.0001^ |
| Sodium (mmol/L) | 137 (IQR: 130 to 144) | | 138 (IQR: 132 to 144) | | 0.02^ |
| Urea (mmol/L) | 6.9 (IQR: 0.6 to 13) | | 6.3 (IQR: 1.5 to 11) | | 1.0 |
| Potassium (mmol/L) | 4.2 (IQR: 3.2 to 5.2) | | N/A | | N/A |
| Operative parameters  (from P-POSSUM) |  | | | | |
| Glasgow coma scale | 15 (IQR: 15 to 15) | | N/A | | N/A |
| Number of procedures | 3 (IQR: 1 to 5) | | 3 (IQR: 1 to 5) | | 0.22 |

Chi-square test used for categorical variables; Mann Whitney test was used for non-parametric continuous dependent variables; ^Statistically significant (p < 0.05)

#### Table S6: Comparison of prognostic factors between developmental and validation data sets: Interventions

| **Prognostic factor** | **Developmental data set**  **(n = 312)** | | **Validation data set**  **(n = 236)** | | **Statistical result** |
| --- | --- | --- | --- | --- | --- |
|  | n | % | n | % | p-value |
| Bowel resection |  | | | | |
| - Before index operation (within same admission) | 52 | 17 | N/A | N/A | N/A |
| - At index operation | 117 | 38 | 96 | 41 | 0.69 |
| - After index operation | 88 | 28 | 55 | 23 | 0.65 |
| CEPOD classification of intervention (emergency cases) | 279 | 89 | 208 | 88 | 1.0 |
| Open abdomen management approach |  | | | | |
| Topical negative pressure (TNP) | 192 | 62 | 179 | 57 | 0.10 |
|  | median value and interquartile range (IQR) | | median value and IQR | | p-value |
| Number of procedures | 3 (IQR: 1 to 5) | | 3 (IQR: 1 to 5) | | 0.22 |
| Length of hospital admission (days)* | 33 (IQR: 0 to 85) | | N/A | | N/A |
| Days abdomen left open | 2 (IQR: 0 to 5) | | 2 (IQR: 0 to 4) | | 0.10 |

Chi-square test used for categorical variables; Mann Whitney test was used for non-parametric continuous dependent variables; ^Statistically significant (p <0.05); *Excludes those that died during index admission
